# Crystal structure of 4-[(*E*)-(2-carbamo­thio­ylhydrazinyl­idene)meth­yl]benzoic acid

**DOI:** 10.1107/S2056989015017594

**Published:** 2015-09-26

**Authors:** Muhammad Nawaz Tahir, Muhammad Anwar-ul-Haq, Muhammad Aziz Choudhary

**Affiliations:** a Department of Physics, University of Sargodha, Sargodha, Punjab, Pakistan; b Department of Chemistry, Mirpur University of Science and Technology (MUST), Mirpur, Azad Jammu and Kashmir, Pakistan

**Keywords:** crystal structure, hydrazinecarbo­thio­amide, hydrogen bonding

## Abstract

The title compound, C_9_H_9_N_3_O_2_S, is close to planar with an r.m.s. deviation of 0.032 Å. An intra­molecular N—H⋯N hydrogen bond closes an *S*(5) ring. In the crystal, mol­ecules are connected into inversion dimers of the *R*_2_^2^(8) type by pairs of O—H⋯O inter­actions. The dimers are further connected by pairs of N—H⋯S inter­actions, which also complete *R*_2_^2^(8) ring motifs. The chains of dimers are cross-linked by N—H⋯O bonds and hence *R*_4_^2^(28) rings are completed. Taken together, these inter­actions lead to infinite sheets propagating in the (122) plane.

## Related literature   

For related structures, see: Carballo *et al.* (2014 ▸); Wu *et al.*, (2009 ▸).
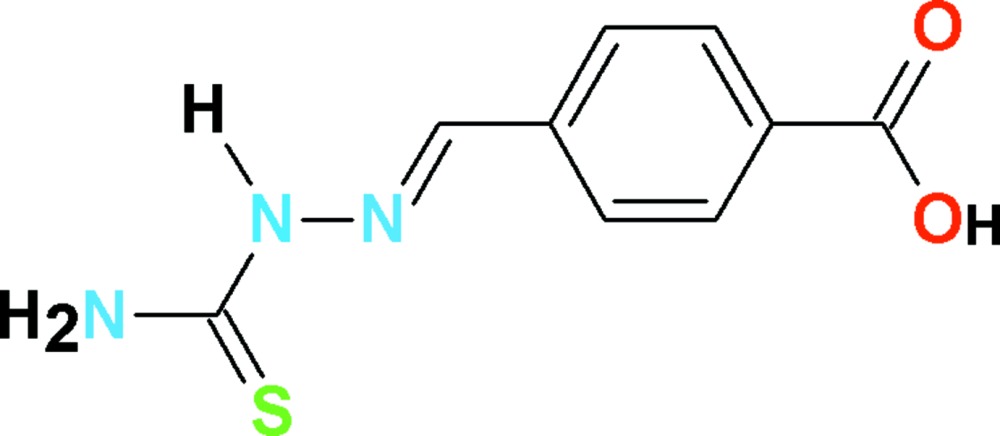


## Experimental   

### Crystal data  

C_9_H_9_N_3_O_2_S
*M* *_r_* = 223.25
Triclinic, 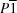

*a* = 4.7454 (5) Å
*b* = 8.5691 (10) Å
*c* = 13.3886 (15) Å
α = 81.386 (6)°
β = 82.878 (6)°
γ = 79.416 (6)°
*V* = 526.52 (10) Å^3^
*Z* = 2
Mo *K*α radiation
μ = 0.29 mm^−1^
*T* = 296 K
0.40 × 0.22 × 0.16 mm

### Data collection  

Bruker Kappa APEXII CCD diffractometer
Absorption correction: multi-scan (*SADABS*; Bruker, 2005 ▸) *T*_min_ = 0.895, *T*_max_ = 0.958
5971 measured reflections
2286 independent reflections
1566 reflections with *I* > 2σ(*I*)
*R*_int_ = 0.030

### Refinement  

*R*[*F* ^2^ > 2σ(*F* ^2^)] = 0.051
*wR*(*F* ^2^) = 0.146
*S* = 1.06
2286 reflections
143 parameters
3 restraints
H atoms treated by a mixture of independent and constrained refinement
Δρ_max_ = 0.55 e Å^−3^
Δρ_min_ = −0.23 e Å^−3^

### 

Data collection: *APEX2* (Bruker, 2007 ▸); cell refinement: *SAINT* (Bruker, 2007 ▸); data reduction: *SAINT*; program(s) used to solve structure: *SHELXS97* (Sheldrick, 2008 ▸); program(s) used to refine structure: *SHELXL2014*/6 (Sheldrick, 2015 ▸); molecular graphics: *ORTEP-3 for Windows* (Farrugia, 2012 ▸) and *PLATON* (Spek, 2009 ▸); software used to prepare material for publication: *WinGX* (Farrugia, 2012 ▸) and *PLATON*.

## Supplementary Material

Crystal structure: contains datablock(s) global, I. DOI: 10.1107/S2056989015017594/hb7508sup1.cif

Structure factors: contains datablock(s) I. DOI: 10.1107/S2056989015017594/hb7508Isup2.hkl

Supporting information file. DOI: 10.1107/S2056989015017594/hb7508Isup3.cml

**Fig. 1.**
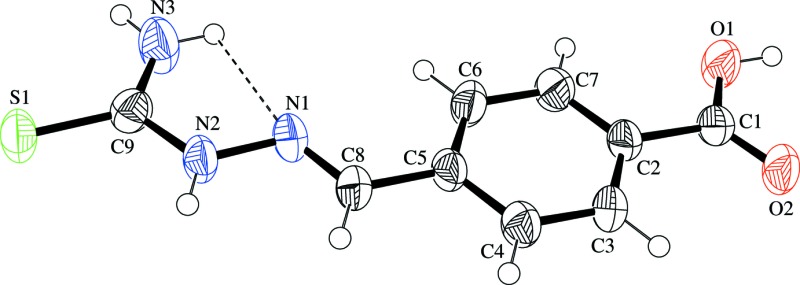
View of the title compound with displacement ellipsoids drawn at the 50% probability level. The dotted line indicate the intramolecular H-interaction.

. DOI: 10.1107/S2056989015017594/hb7508fig1.tif
View of the title compound with displacement ellipsoids drawn at the 50% probability level. The dotted line indicate the intra­molecular H-inter­action.

**Fig. 2.**
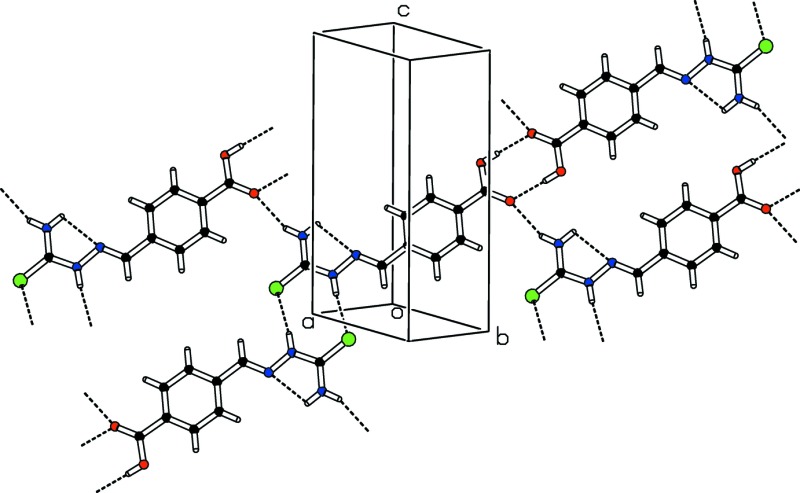
The partial packing (PLATON; Spek, 2009), which shows that molecules are dimerized and form a two-dimensional network with various ring motifs.

PLATON . DOI: 10.1107/S2056989015017594/hb7508fig2.tif
The partial packing (*PLATON*; Spek, 2009), which shows that mol­ecules are dimerized and form a two-dimensional network with various ring motifs.

CCDC reference: 1426244

Additional supporting information:  crystallographic information; 3D view; checkCIF report

## Figures and Tables

**Table 1:** Hydrogen-bond geometry (, )

*D*H*A*	*D*H	H*A*	*D* *A*	*D*H*A*
N3H3*B*N1	0.86(3)	2.28(3)	2.600(3)	102(2)
O1H1O2^i^	0.82	1.84	2.653(2)	170
N2H2S1^ii^	0.86	2.53	3.347(2)	160
N3H3*A*O2^iii^	0.85(1)	2.14(2)	2.918(3)	152(3)

Symmetry codes: (i) 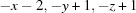; (ii) 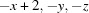; (iii) 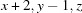.

## supplementary crystallographic information

### S1. Comment

The title compound (I, Fig. 1) has been synthesized for the complexation and other studies. The crystal structures of 2-(4-cyanobenzylidene)hydrazinecarbothioamide (Wu *et al.*, 2009) and 2-(4-formylbenzylidene)hydrazinecarbothioamide (Carballo *et al.*, 2014) have been published which are related to (I).

The heavy atoms of (I) i.e (C1–C9/N1/M2/M3/O1/O2/S1) are almost in a plane. The r. m. s. deviation from the mean square plane is 0.0312 Å. *S* (5) ring motif is present due to intramolecular H-interaction of N–H···N type (Table 1, Fig. 1). The molecules are dimerzed due to conventional O–H···O interaction with *R*_2_^2^(8) rings (Table 1, Fig. 2). These dimmers are connected from opposite ends due to N–H···S interactions and also complete *R*_2_^2^(8) ring motifs. *R*_4_^2^(28) rings are completed (Table 1, Fig. 2), when we consider N–H···O and O–H···O contacts (Table 1, Fig. 2). The molecules are overall stabilized in the form of a two dimensional network with base vectors [4 - 1 -1] and [2 - 1 0] in the (1 2 2) plane.

### S2. Experimental

Equimolar quantities of 4-formylbenzoic acid and thiosemicarbazide were dissolved separately in methanol and then mixed. The mixture was refluxed for 3 h. The resulting solution was kept at room temperature for crystallization which afforded white needle after 48 h. m.p. 455 K

### S3. Refinement

The coordinates of H-atoms of NH_2_ group were refined with constraints. The other H-atoms were positioned geometrically (C–H = 0.93 Å, N–H = 0.86 Å, O–H = 0.82 Å)and refined as riding with *U*_iso_(H) = *xU*_eq_(C, N O), where *x* = 1.5 for hydroxy and NH_2_ groups and *x* = 1.2 for other H-atoms.

### Crystal data

**Table:**

C_9_H_9_N_3_O_2_S	*Z* = 2
*M**_r_* = 223.25	*F*(000) = 232
Triclinic, *P*1	*D*_x_ = 1.408 Mg m^−^^3^
*a* = 4.7454 (5) Å	Mo *K*α radiation, λ = 0.71073 Å
*b* = 8.5691 (10) Å	Cell parameters from 1566 reflections
*c* = 13.3886 (15) Å	θ = 1.6–27.0°
α = 81.386 (6)°	µ = 0.29 mm^−^^1^
β = 82.878 (6)°	*T* = 296 K
γ = 79.416 (6)°	Needle, colourless
*V* = 526.52 (10) Å^3^	0.40 × 0.22 × 0.16 mm

### Data collection

**Table:**

Bruker Kappa APEXII CCD diffractometer	2286 independent reflections
Radiation source: fine-focus sealed tube	1566 reflections with *I* > 2σ(*I*)
Graphite monochromator	*R*_int_ = 0.030
Detector resolution: 7.70 pixels mm^-1^	θ_max_ = 27.0°, θ_min_ = 1.6°
ω scans	*h* = −5→6
Absorption correction: multi-scan (*SADABS*; Bruker, 2005)	*k* = −10→10
*T*_min_ = 0.895, *T*_max_ = 0.958	*l* = −17→17
5971 measured reflections	

### Refinement

**Table:**

Refinement on *F*^2^	Primary atom site location: structure-invariant direct methods
Least-squares matrix: full	Secondary atom site location: difference Fourier map
*R*[*F*^2^ > 2σ(*F*^2^)] = 0.051	Hydrogen site location: inferred from neighbouring sites
*wR*(*F*^2^) = 0.146	H atoms treated by a mixture of independent and constrained refinement
*S* = 1.06	*w* = 1/[σ^2^(*F*_o_^2^) + (0.0687*P*)^2^ + 0.105*P*] where *P* = (*F*_o_^2^ + 2*F*_c_^2^)/3
2286 reflections	(Δ/σ)_max_ < 0.001
143 parameters	Δρ_max_ = 0.55 e Å^−^^3^
3 restraints	Δρ_min_ = −0.23 e Å^−^^3^

### Special details

**Table:**

Geometry. All e.s.d.'s (except the e.s.d. in the dihedral angle between two l.s. planes) are estimated using the full covariance matrix. The cell e.s.d.'s are taken into account individually in the estimation of e.s.d.'s in distances, angles and torsion angles; correlations between e.s.d.'s in cell parameters are only used when they are defined by crystal symmetry. An approximate (isotropic) treatment of cell e.s.d.'s is used for estimating e.s.d.'s involving l.s. planes.
Refinement. Refinement of *F*^2^ against ALL reflections. The weighted *R*-factor *wR* and goodness of fit *S* are based on *F*^2^, conventional *R*-factors *R* are based on *F*, with *F* set to zero for negative *F*^2^. The threshold expression of *F*^2^ > σ(*F*^2^) is used only for calculating *R*-factors(gt) *etc*. and is not relevant to the choice of reflections for refinement. *R*-factors based on *F*^2^ are statistically about twice as large as those based on *F*, and *R*-factors based on ALL data will be even larger.

### Fractional atomic coordinates and isotropic or equivalent isotropic displacement parameters (Å^2^)

**Table:**

	*x*	*y*	*z*	*U*_iso_*/*U*_eq_	
S1	1.16213 (14)	−0.24283 (8)	0.07250 (5)	0.0516 (3)	
O1	−0.7098 (4)	0.3266 (3)	0.50791 (14)	0.0659 (6)	
H1	−0.8389	0.3791	0.5417	0.099*	
O2	−0.8294 (4)	0.5276 (2)	0.38826 (14)	0.0598 (6)	
N1	0.4804 (4)	0.0209 (2)	0.19255 (16)	0.0417 (5)	
N2	0.7068 (4)	−0.0327 (2)	0.12477 (16)	0.0426 (5)	
H2	0.7307	0.0194	0.0651	0.051*	
N3	0.8427 (6)	−0.2352 (3)	0.24762 (19)	0.0635 (7)	
H3A	0.951 (6)	−0.321 (3)	0.270 (2)	0.095*	
H3B	0.702 (5)	−0.199 (4)	0.289 (2)	0.095*	
C1	−0.6710 (5)	0.4042 (3)	0.41755 (19)	0.0443 (6)	
C2	−0.4179 (5)	0.3333 (3)	0.35245 (19)	0.0409 (6)	
C3	−0.3616 (5)	0.4082 (3)	0.2550 (2)	0.0426 (6)	
H3	−0.4830	0.5005	0.2308	0.051*	
C4	−0.1243 (5)	0.3456 (3)	0.1935 (2)	0.0434 (6)	
H4	−0.0858	0.3969	0.1282	0.052*	
C5	0.0572 (4)	0.2066 (3)	0.22859 (19)	0.0380 (6)	
C6	−0.0038 (5)	0.1310 (3)	0.3255 (2)	0.0488 (7)	
H6	0.1146	0.0372	0.3493	0.059*	
C7	−0.2402 (5)	0.1942 (3)	0.3875 (2)	0.0495 (7)	
H7	−0.2795	0.1430	0.4527	0.059*	
C8	0.3065 (5)	0.1441 (3)	0.16108 (19)	0.0406 (6)	
H8	0.3370	0.1949	0.0952	0.049*	
C9	0.8922 (5)	−0.1683 (3)	0.15277 (19)	0.0415 (6)	

### Atomic displacement parameters (Å^2^)

**Table:**

	*U*^11^	*U*^22^	*U*^33^	*U*^12^	*U*^13^	*U*^23^
S1	0.0457 (4)	0.0493 (4)	0.0491 (4)	0.0093 (3)	0.0097 (3)	−0.0059 (3)
O1	0.0503 (11)	0.0888 (16)	0.0404 (11)	0.0235 (10)	0.0101 (8)	−0.0059 (11)
O2	0.0497 (11)	0.0690 (13)	0.0463 (11)	0.0202 (10)	0.0079 (8)	−0.0087 (10)
N1	0.0345 (10)	0.0446 (12)	0.0429 (12)	−0.0032 (9)	0.0093 (9)	−0.0108 (10)
N2	0.0366 (10)	0.0457 (12)	0.0378 (12)	0.0032 (9)	0.0092 (8)	−0.0041 (9)
N3	0.0702 (17)	0.0557 (16)	0.0458 (15)	0.0152 (12)	0.0145 (12)	0.0054 (12)
C1	0.0340 (13)	0.0574 (17)	0.0390 (15)	0.0029 (12)	−0.0009 (10)	−0.0133 (13)
C2	0.0306 (12)	0.0519 (15)	0.0393 (14)	0.0012 (10)	0.0003 (10)	−0.0160 (12)
C3	0.0330 (12)	0.0452 (14)	0.0461 (15)	0.0034 (10)	0.0014 (10)	−0.0115 (12)
C4	0.0381 (13)	0.0483 (15)	0.0402 (14)	−0.0027 (11)	0.0046 (10)	−0.0069 (12)
C5	0.0273 (11)	0.0465 (14)	0.0407 (14)	−0.0036 (10)	0.0014 (9)	−0.0143 (11)
C6	0.0382 (13)	0.0536 (16)	0.0466 (16)	0.0088 (11)	0.0018 (11)	−0.0060 (13)
C7	0.0419 (14)	0.0632 (18)	0.0359 (14)	0.0025 (12)	0.0048 (11)	−0.0039 (13)
C8	0.0324 (12)	0.0458 (14)	0.0406 (14)	0.0007 (10)	0.0023 (10)	−0.0094 (12)
C9	0.0382 (13)	0.0395 (14)	0.0444 (15)	−0.0050 (10)	−0.0033 (10)	−0.0007 (12)

### Geometric parameters (Å, º)

**Table:**

S1—C9	1.664 (2)	C2—C3	1.383 (4)
O1—C1	1.299 (3)	C2—C7	1.383 (3)
O1—H1	0.8200	C3—C4	1.383 (3)
O2—C1	1.222 (3)	C3—H3	0.9300
N1—C8	1.268 (3)	C4—C5	1.392 (3)
N1—N2	1.375 (3)	C4—H4	0.9300
N2—C9	1.357 (3)	C5—C6	1.383 (4)
N2—H2	0.8600	C5—C8	1.466 (3)
N3—C9	1.325 (3)	C6—C7	1.386 (3)
N3—H3A	0.853 (10)	C6—H6	0.9300
N3—H3B	0.851 (10)	C7—H7	0.9300
C1—C2	1.484 (3)	C8—H8	0.9300
			
C1—O1—H1	109.5	C3—C4—H4	119.7
C8—N1—N2	116.9 (2)	C5—C4—H4	119.7
C9—N2—N1	119.5 (2)	C6—C5—C4	119.1 (2)
C9—N2—H2	120.3	C6—C5—C8	122.0 (2)
N1—N2—H2	120.3	C4—C5—C8	118.8 (2)
C9—N3—H3A	120 (2)	C5—C6—C7	120.4 (2)
C9—N3—H3B	124 (2)	C5—C6—H6	119.8
H3A—N3—H3B	116 (3)	C7—C6—H6	119.8
O2—C1—O1	122.6 (2)	C2—C7—C6	120.1 (2)
O2—C1—C2	122.2 (2)	C2—C7—H7	119.9
O1—C1—C2	115.2 (2)	C6—C7—H7	119.9
C3—C2—C7	119.9 (2)	N1—C8—C5	120.0 (2)
C3—C2—C1	118.9 (2)	N1—C8—H8	120.0
C7—C2—C1	121.2 (2)	C5—C8—H8	120.0
C4—C3—C2	119.9 (2)	N3—C9—N2	115.3 (2)
C4—C3—H3	120.0	N3—C9—S1	123.1 (2)
C2—C3—H3	120.0	N2—C9—S1	121.62 (19)
C3—C4—C5	120.5 (2)		
			
C8—N1—N2—C9	−177.0 (2)	C4—C5—C6—C7	−0.9 (4)
O2—C1—C2—C3	−0.8 (4)	C8—C5—C6—C7	179.5 (2)
O1—C1—C2—C3	179.8 (2)	C3—C2—C7—C6	0.9 (4)
O2—C1—C2—C7	179.5 (3)	C1—C2—C7—C6	−179.5 (2)
O1—C1—C2—C7	0.1 (4)	C5—C6—C7—C2	0.3 (4)
C7—C2—C3—C4	−1.3 (4)	N2—N1—C8—C5	179.0 (2)
C1—C2—C3—C4	179.0 (2)	C6—C5—C8—N1	−2.9 (4)
C2—C3—C4—C5	0.7 (4)	C4—C5—C8—N1	177.6 (2)
C3—C4—C5—C6	0.5 (4)	N1—N2—C9—N3	−2.7 (4)
C3—C4—C5—C8	180.0 (2)	N1—N2—C9—S1	177.48 (17)

### Hydrogen-bond geometry (Å, º)

**Table:**

*D*—H···*A*	*D*—H	H···*A*	*D*···*A*	*D*—H···*A*
N3—H3*B*···N1	0.86 (3)	2.28 (3)	2.600 (3)	102 (2)
O1—H1···O2^i^	0.82	1.84	2.653 (2)	170
N2—H2···S1^ii^	0.86	2.53	3.347 (2)	160
N3—H3*A*···O2^iii^	0.85 (1)	2.14 (2)	2.918 (3)	152 (3)

Symmetry codes: (i) −*x*−2, −*y*+1, −*z*+1; (ii) −*x*+2, −*y*, −*z*; (iii) *x*+2, *y*−1, *z*.
