# Supplementary figures and images for: Crystal structure of 4-[(E)-(2-carbamo­thio­ylhydrazinyl­idene)meth­yl]benzoic acid
Source: Acta Crystallogr E Crystallogr Commun. 2015 Sep 26;71(Pt 10):o772. doi: 10.1107/S2056989015017594 (PMC4647436; doi:10.1107/S2056989015017594)

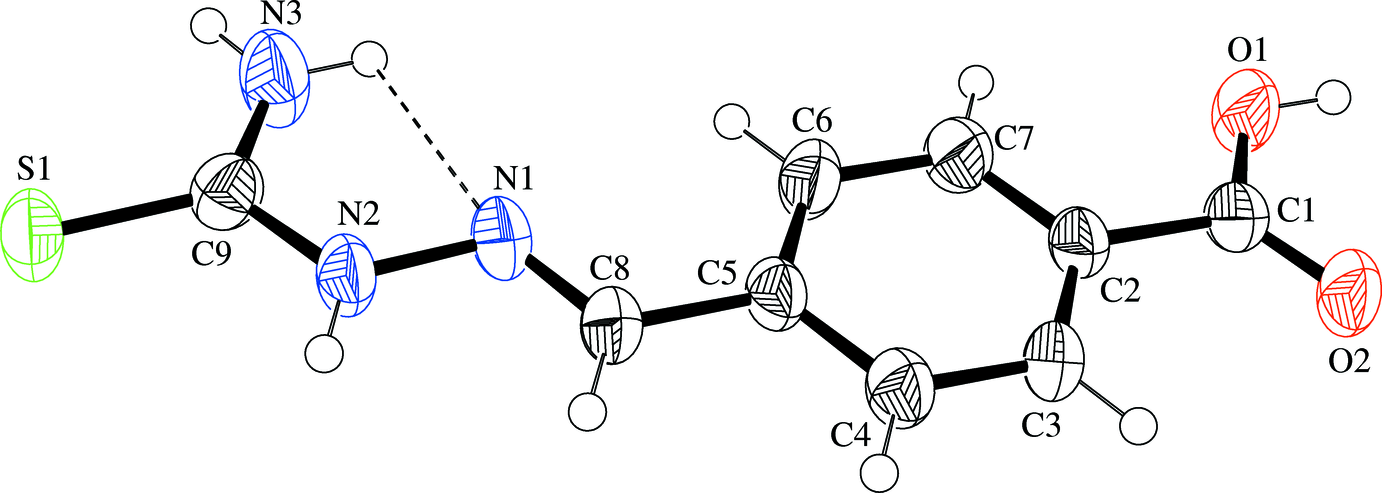

Supplement: Supplementary file 4 [file e-71-0o772-fig1.tif]

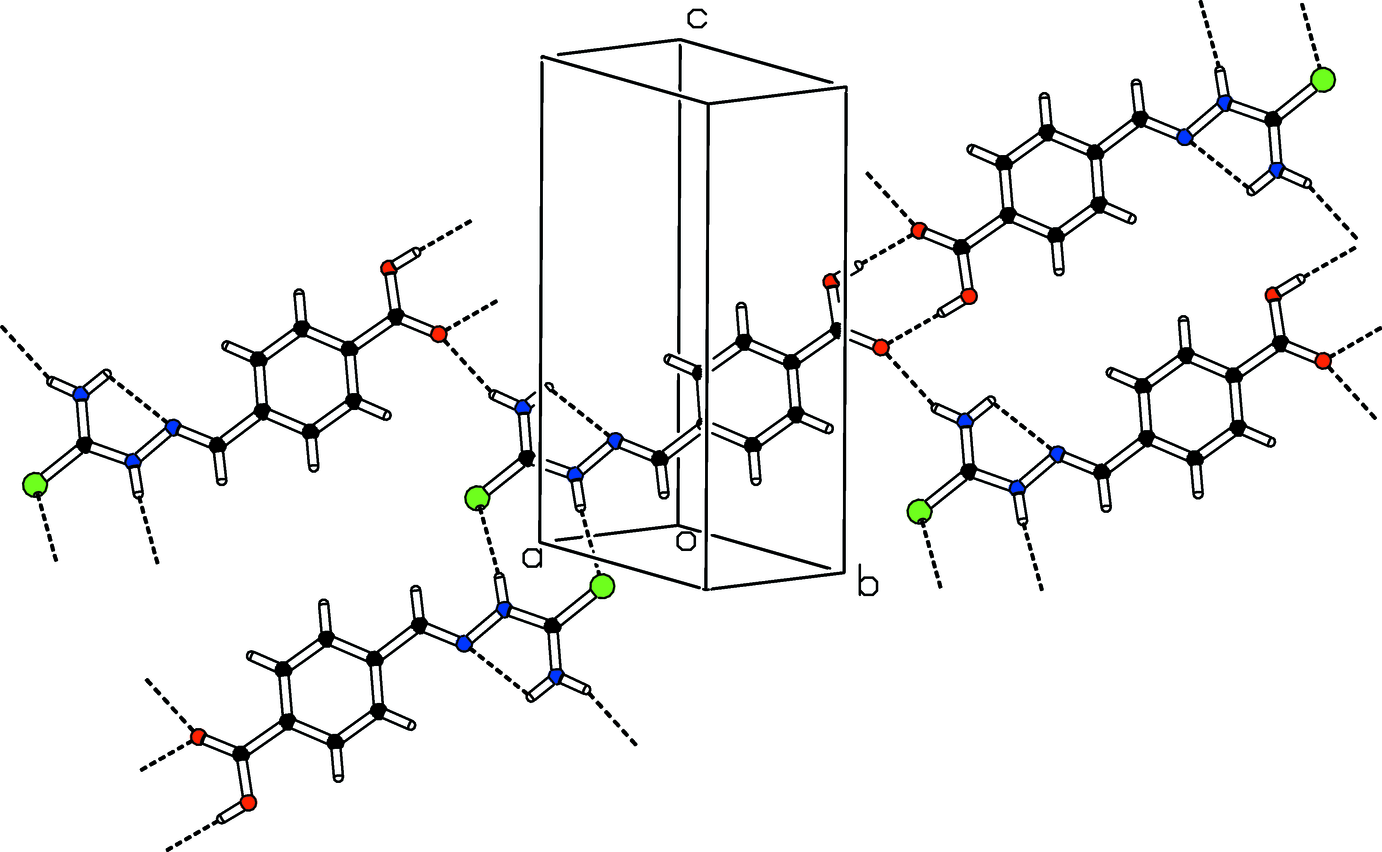

Supplement: Supplementary file 5 [file e-71-0o772-fig2.tif]
